# Sesame Oil Attenuates Ovalbumin-Induced Pulmonary Edema and Bronchial Neutrophilic Inflammation in Mice

**DOI:** 10.1155/2013/905670

**Published:** 2013-04-04

**Authors:** Dur-Zong Hsu, Chuan-Teng Liu, Pei-Yi Chu, Ya-Hui Li, Srinivasan Periasamy, Ming-Yie Liu

**Affiliations:** ^1^Department of Environmental and Occupational Health, National Cheng Kung University Medical College, 138 Sheng-Li Road, Tainan 70428, Taiwan; ^2^Sustainable Environment Research Center, National Cheng Kung University, Tainan 70428, Taiwan

## Abstract

*Background*. Allergic asthma is one of the most common chronic inflammatory diseases of airways. Severe asthma may lead to hospitalization and death. Sesame oil is a natural product with anti-inflammatory property. However, the effect of sesame oil on allergic asthma has never been studied. *Objective*. We investigate the effect of sesame oil on pulmonary inflammation in allergic asthma model. *Methods*. Allergic airway inflammation was induced by sensitizing with two doses of 10 mg ovalbumin (OVA) and then challenged with 1% OVA nebulizer exposure (1 h/day) for 3 days. Sesame oil (0.25, 0.5, or 1 mL/kg/day) was given orally 30 min before each challenge. Samples were collected 24 h after the last challenge. *Results*. Data showed that sesame oil inhibited pulmonary edema and decreased interleukin (IL)-1**β** and IL-6 levels in bronchoalveolar lavage fluid in OVA-treated mice. Sesame oil also decreased pulmonary nitrite level, inducible nitric oxide synthase expression, and neutrophil infiltration induced by OVA. Further, sesame oil decreased serum IgE level in OVA-treated mice. *Conclusion*. Sesame oil may attenuate pulmonary edema and bronchial neutrophilic inflammation by inhibiting systemic IgE level in allergic asthma.

## 1. Introduction

Allergic asthma is a chronic inflammatory lung disease of airways, which affects more than 22 million people in the United States. Despite advanced understanding of the pathophysiology of asthma, affected patients continue to incur significant morbidity from the disease [1]. Patients with this allergy who also consistently have very poorly controlled asthma were more likely to have a corticosteroid burst or be subjected to hospitalization or an emergency department visit [2].

Airway edema constitutes one of the key features of allergic airway inflammation [3]. Proinflammatory cytokine, such as interleukin-1 and 6, overproduction is involved in the pathogenesis of airway inflammation [4, 5]. Nitric oxide (NO) in asthma has been recently used as a biomarker for asthma inflammation [6]. The high concentrations of NO in asthma may produce various deleterious effects including increased vascular permeability, damage to the airway epithelium, and promotion of inflammatory response by reregulating proinflammatory cytokine generation [7]. In addition, leukocyte (such as neutrophil) infiltration plays a crucial role in pulmonary inflammation in allergic asthma [8–10]. Neutrophils and other leukocytes adhere via cognate receptors to the pulmonary endothelium. Activated neutrophils release proteases, leukotrienes, reactive oxygen intermediates, and other inflammatory molecules that amplify the inflammatory response [11]. In addition, IgE plays a pivotal role in the propagation of airway inflammation in allergic asthma. In humans, IgE levels positively correlate with the presence of asthma symptoms, probability for allergic sensitization, and emergency room visits [12].

Sesame oil is derived from the plant species *Sesamum indicum *L., a herbaceous annual belonging to the Pedaliaceae family [13]. The main constituents of sesame oil include fatty acids, lignans, and antioxidants, such as *γ*-tocopherol [14]. The fatty acids in sesame oil include palmitic acid (16 : 0; 7–12%), palmitoleic acid (16 : 1; <0.5%), stearic acid (18 : 0; 3.5–6%), oleic acid (18 : 1; 35–50%), linoleic acid (18 : 2; 35–50%), linolenic acid (18 : 3; <1.0%), and eicosanoic acid (20 : 1; <1.0%). Sesame oil has been reported to have potent anti-inflammatory effect on various models, such as endotoxemia [15] and heavy metal poisoning [16]. However, the effect of sesame oil on allergic asthma has never been investigated. In the present study, we examined the protective effect of sesame oil on pulmonary edema and inflammation by using a mouse model of allergic asthma.

## 2. Materials and Methods

### 2.1. Animals

Female BALB/c mice weighing 25–30 g were purchased from and housed in our institution's laboratory animal center. The mice were housed individually in a room with a 12-hour light/dark cycle and central air conditioning (25°C, 70% humidity). They were allowed free access to tap water and were given a rodent diet (Richmond Standard, PMI Feeds, Inc., St. Louis, MO, USA). The animal care and experimental protocols were in accord with nationally approved guidelines.

### 2.2. Experimental Design

**Figure 1:**
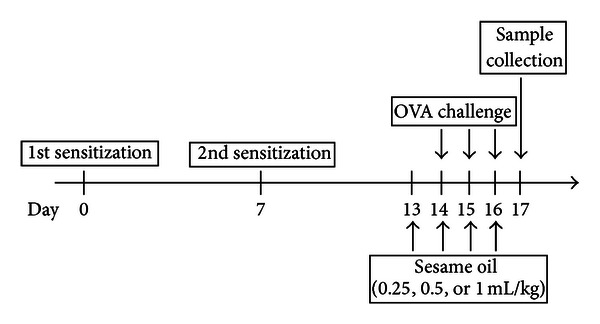
The experimental design of OVA-induced allergic asthma mice model.

To induce allergic airway inflammation, mice were sensitized with an intraperitoneal injection of 10 mg ovalbumin (OVA, albumin chicken egg, Sigma A-5378) plus 1 mg aluminum hydroxide (Imject Alum, Thermo) on day 0 and 7. Control mice received an injection of phosphate-buffered saline (PBS) with aluminum hydroxide. Then, mice were challenged with the exposure of 1% OVA in PBS through ultrasonic nebulizer (NE-U17, Omron) with a regulated flow rate for 1 h/day from day 14 to day 16 [1, 17]. Sesame oil (0.25, 0.5, or 1 mL/kg/day; orally) was given 30 min from day 13 to 16 before challenge. Serum samples, bronchoalveolar lavage fluid (BALF), and lung tissue of the mice were collected on day 17 (Figure 1).

### 2.3. Assessing Lung Edema

Lungs were blotted on gauze to remove excess fluid, and wet weights were recorded immediately. The lungs were then dried for 72 h at 90°C, and weights of the dried lungs were recorded. Data are presented as the ratio of wet lung weight relative to dry lung weight, as we previously described [18]. 

### 2.4. BALF Cytokine Measurement

Interleukin (IL)-1*β* and IL-6 levels were quantified in the cell-free supernatant of BALF by using commercial enzyme-linked immunosorbent assay (ELISA) kits (Duo-Set; R&D Systems Inc., Minneapolis, MN, USA). In brief, a 96-well immunoassay plate was coated with capture-antibody (100 *μ*L/well) overnight at room temperature, followed by a blocking step. Recombinant cytokines ranging from 7.81 to 1000 pg/mL were used as standards. One hundred microliters of test sample and serial standards were incubated at room temperature for 2 h. After samples were incubated with 100 *μ*L of biotinylated rabbit anti-mouse IL-1*β* or IL-6 antibody, streptavidin-conjugated horseradish peroxidase was added for 20 min at room temperature. The peroxidase reaction was initiated by the addition of 100 *μ*L of 3,3′,5,5′-tetramethylbenzidine/H_2_O_2_ (R&D systems) for 30 min and then stopped by addition of 50 *μ*L of 0.5 M H_2_SO_4_. The absorbance was measured at 450 nm with an ELISA reader [19]. 

### 2.5. Measurement of Pulmonary Nitrite Concentration

Briefly, nitrite levels in lung tissue were measured by Griess reaction. Lung tissue was homogenized in deionized water (1 : 10, wt/vol). Tissue homogenate (500 *μ*L) was centrifuged at 2,500 × g for 10 min at 4°C. Supernatant (100 *μ*L) was incubated with 100 *μ*L of Griess reagent at room temperature for 20 min. The absorbance was measured at 550 nm using a spectrophotometer (DU 640B; Beckman Coulter Inc., Fullerton, CA., USA). Nitrite concentration was calculated by comparing it with a standard solution of known sodium nitrite concentration [20].

### 2.6. Western Blotting

We loaded 60 *μ*g of protein on 10% sodium dodecyl sulfate-polyacrylamide gel for electrophoresis and then transferred it to nitrocellulose sheets (NEN Life Science Products, Inc., Boston, MA, USA) in a transfer apparatus (Bio-Rad) run at 1.2 A for 3 h. After the blots had been blocked in 5% nonfat skim milk in Tris-buffered saline Tween-20, we incubated the blots with primary inducible NO synthase (iNOS) polyclonal antibody (dilution, 1 : 1,000; BD Biosciences, San Diego, CA, USA) against the target protein in 5% nonfat skim milk and then with anti-rabbit immunoglobulin G conjugated with alkaline phosphatase (dilution, 1 : 3,000; Jackson ImmunoResearch Laboratories, Inc., Philadelphia, PA, USA). Immunoblots were developed using alkaline phosphatase substrate solution (5-bromo-4-chloro-3-indolyl-phosphate/nitroblue tetrazolium; Kirkegaard and Perry Laboratories, Inc., Baltimore, MD, USA).

### 2.7. BALF Collection and Total Cell Counting

The lung was lavaged with a total of 4 mL of sterile PBS by using a 1 mL syringe with a 22G needle inserted into the trachea, and BALF was collected through 4-time-instilled and withdrawn. BAL cells were collected by centrifugation at 1000 rpm for 10 min at 4°C. The cell pellet was resuspended, and viable cells were counted by using trypan blue dye exclusion.

### 2.8. Semiquantitative Scoring of the Inflammatory Changes in Lung

Semiquantitative scoring of the inflammatory changes was performed as described by Zeldin et al. [21] and Elekes et al., [22] on the basis of the presence or abundance of the following: (1) perivascular oedema (0: absent; 1: mild to moderate, involving less than 25% of the perivascular spaces; 2: moderate to severe, involving more than 25% but less than 75% of perivascular spaces; 3: severe, involving more than 75% of perivascular spaces); (2) perivascular/peribronchial acute inflammation (0: absent; 1: mild acute inflammation in the perivascular oedematous space with fewer than 5 neutrophils per high-power field; 2: moderate acute inflammation in the perivascular spaces extending to involve the peribronchial spaces with more than 5 neutrophils per high-power field in these regions; 3: severe acute inflammation in the perivascular and peribronchial spaces with numerous neutrophils around most bronchioles); (3) macrophages/mononuclear cells in the alveolar spaces (0: absent; 1: present in fewer than 25% of alveolar spaces; 2: >25% of alveolar spaces). The score values for these individual parameters were added to form a composite inflammatory score ranging from 0 to 10. From every specimen, 6–8 sections were taken from different depths to give a representative appreciation of the whole lung. Mean scores were determined from the different sections of the individual animals, and composite score values of the different experimental groups were calculated from these mean scores.

### 2.9. Histological Evaluation of Lung Inflammation

A histological examination was used to assess lung inflammation. Briefly, organ tissue was fixed in 4% formaldehyde buffered with a phosphate solution (0.1 M, pH 7.4) at room temperature. Organ fragments were washed in phosphate buffer, dehydrated in graded concentrations of ethanol, and then embedded in paraffin. From each tissue, 4 *μ*m thin sections were obtained and stained with hematoxylin and eosin to evaluate lung morphology and then mounted using Depex Polystyrene dissolved in xylene mountant. The permanently mounted sections of lung tissue were examined under a microscope (Eclipse E 600; Nikon Instech Co Ltd, Kawasaki, Japan; 100x magnification) to assess pulmonary injury.

### 2.10. Serum Total IgE Measurement

Serum IgE levels were measured quantitatively using commercially available ELISA kits (BETHYL E90-115). In brief, a 96-well immunoassay plate was coated with capture-antibody (100 *μ*L/well) overnight at room temperature, followed by a blocking step. Recombinant IgE levels ranging from 1.95 to 250 ng/mL were used as standards. One hundred microliters of test sample and serial standards were incubated at room temperature for 2 h. After serum samples were incubated with 100 *μ*L of biotinylated rabbit anti-mouse IgE antibody, streptavidin-conjugated horseradish peroxidase was added for 20 min at room temperature. The peroxidase reaction was initiated by the addition of 100 *μ*L of 3,3′,5,5′-tetramethylbenzidine/H_2_O_2_ (R&D systems) for 30 min and then stopped by addition of 50 *μ*L of 0.5 M H_2_SO_4_. The absorbance was measured at 450 nm with an ELISA reader. 

### 2.11. Statistical Analysis

Data are mean ± standard deviation (SD). Significant differences between measurements traits were analyzed using one-way ANOVA followed by Student *t*-test method. Statistical significance was set at *P* < 0.05.

## 3. Results

### 3.1. Effects of Sesame Oil on Pulmonary Edema in OVA-Treated Mice

**Figure 2:**
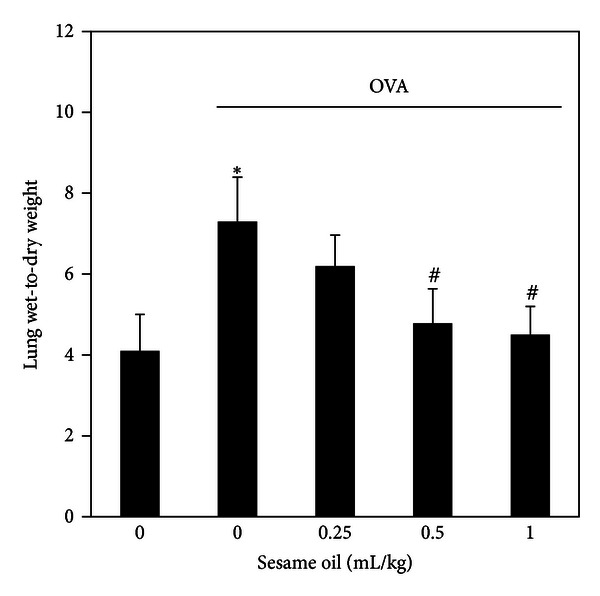
The effect of sesame oil on lung edema in OVA-treated mice. Sesame oil (0, 0.25, 0.5, or 1 mL/kg, orally) was given to OVA-treated mice and lung wet-to-dry weight was assessed. Data were mean ± SD (*n* = 8). Significant differences of measurement traits were analyzed using one-way ANOVA followed by Students' *t*-test. *P < 0.05 compared with control group; ^#^ *P* < 0.05 compared with OVA-alone group.

To examine the effects of sesame oil on allergic asthma-associated airway inflammation, pulmonary edema was assessed. The lung wet-to-dry weight of mice treated with OVA was significantly increased (7.3 ± 1.1) compared with control group (4.1 ± 0.9). Sesame oil (0.5 and 1 mL/kg) decreased the wet-to-dry weight (4.8 ± 0.8 and 4.5 ± 0.7, resp.) in OVA-treated mice (Figure 2).

### 3.2. Effects of Sesame Oil on Pulmonary Inflammatory Cytokine Production in OVA-Treated Mice

**Figure 3:**
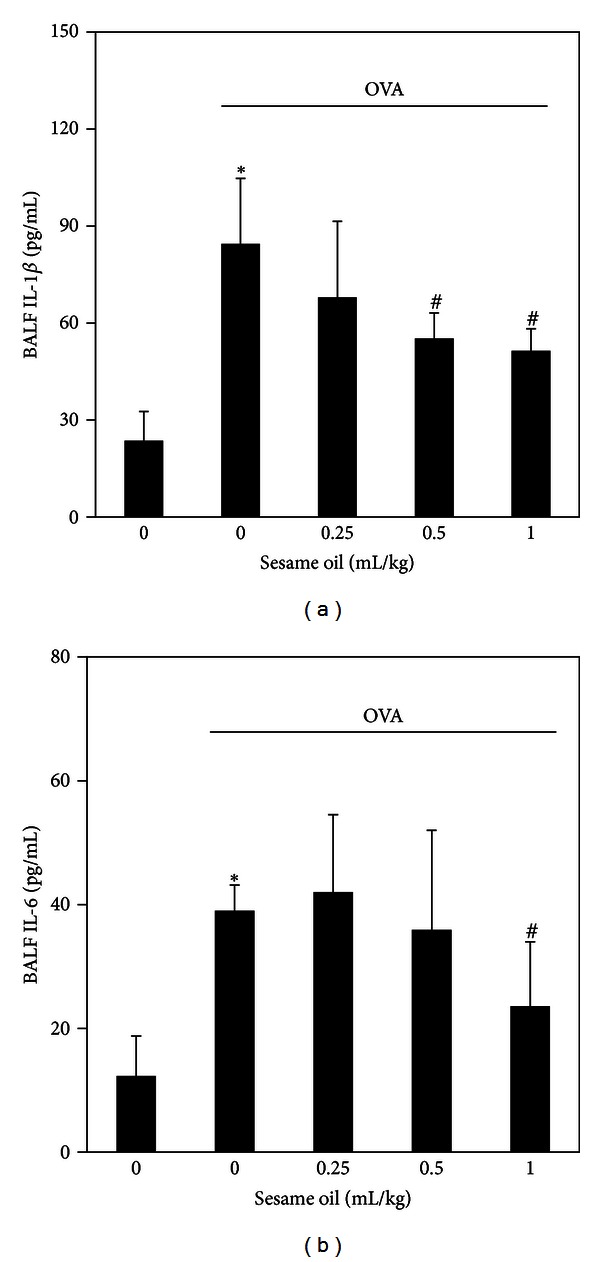
The effect of sesame oil on proinflammatory cytokines production in lungs in OVA-treated mice. Sesame oil (0, 0.25, 0.5, or 1 mL/kg, orally) was given to OVA-treated mice and IL-1*β* (a) and IL-6 (b) levels in BALF were determined. Data were mean ± SD (*n* = 8). Significant differences of measurement traits were analyzed using one-way ANOVA followed by Students' *t*-test. *P < 0.05 compared with control group; ^#^ *P* < 0.05 compared with OVA-alone group.

To examine the involvement of inflammatory cytokine in sesame oil's protection against allergic asthma, BALF IL-1*β* and IL-6 levels were determined. Sesame oil significantly decreased OVA-induced IL-1*β* (51.47 ± 6.74) (Figure 3(a)) and IL-6 (23.60 ± 10.37) (Figure 3(b)) productions compared with the OVA-alone group IL-1*β* (84.51 ± 20.23) (Figure 3(a)) and IL-6 (39.03 ± 4.11) (Figure 3(b)).

### 3.3. Effects of Sesame Oil on Pulmonary Nitric Oxide Production and iNOS Expression in OVA-Treated Mice

**Figure 4:**
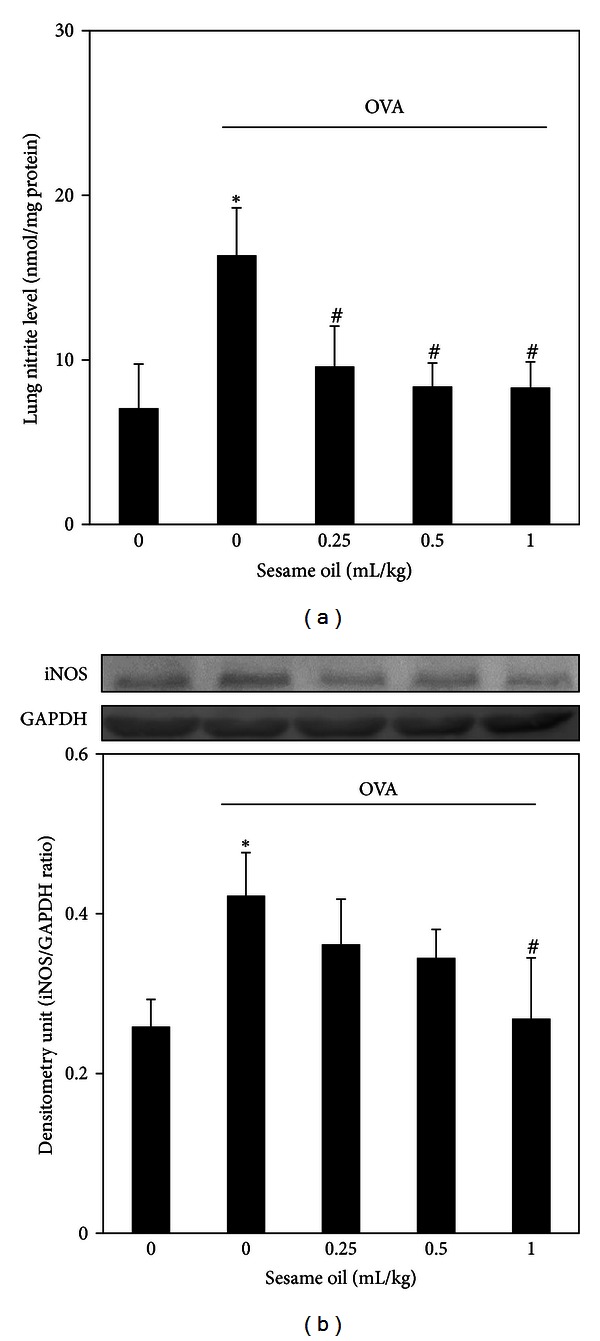
The effect of sesame oil on nitric oxide production and iNOS expression in lungs in OVA-treated mice. Sesame oil (0, 0.25, 0.5, or 1 mL/kg, orally) was given to OVA-treated mice and lung nitrite level (a) and iNOS expression (b) were determined. Data were mean ± SD (*n* = 8). Significant differences of measurement traits were analyzed using one-way ANOVA followed by Students' *t*-test. *P < 0.05 compared with control group; ^#^ *P* < 0.05 compared with OVA-alone group.

To examine the involvement of nitric oxide in sesame oil's protection against allergic asthma, nitrite level and iNOS expression in lung tissue were determined. Sesame oil significantly decreased OVA-induced nitrite production (8.32 ± 1.57) (Figure 4(a)) and iNOS expression (0.27 ± 0.08) (Figure 4(b)) compared with the OVA-alone group nitrite production (16.36 ± 2.87) (Figure 4(a)) and iNOS expression (0.42 ± 0.053) (Figure 4(b)).

### 3.4. Effects of Sesame Oil on Inflammatory Cell Infiltration in OVA-Treated Mice

**Figure 5:**
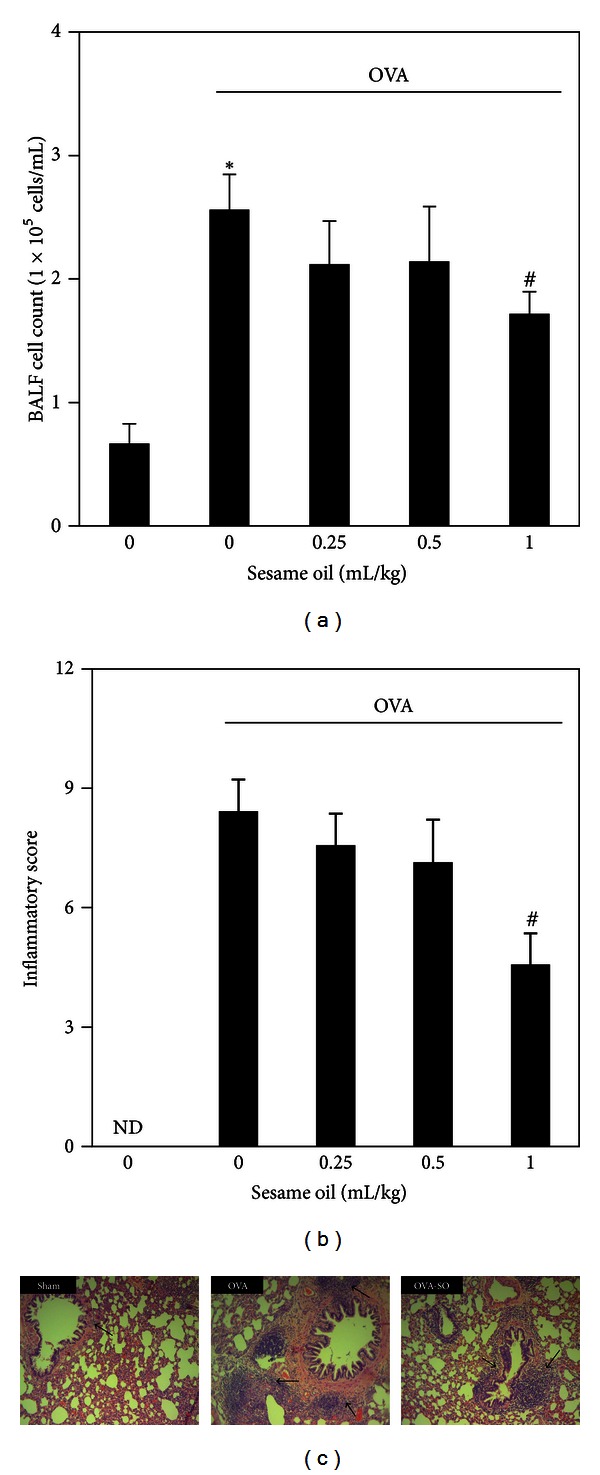
The effect of sesame oil on inflammatory cell infiltration in lungs in OVA-treated mice. Sesame oil (0, 0.25, 0.5, or 1 mL/kg, orally) was given to OVA-treated mice, and BALF cell count (a), inflammatory score (b), and tissue histological examination (c) were assessed. Data were mean ± SD (*n* = 8). Significant differences of measurement traits were analyzed using one-way ANOVA followed by Students' *t*-test. *P < 0.05 compared with control group; ^#^ *P* < 0.05 compared with OVA-alone group. ND: nondetected. Sham group: mice treated with saline. OVA group: mice treated with OVA only. OVA-SO group: mice treated with sesame oil plus OVA. The arrows indicate neutrophil infiltration (hematoxylin and eosin stain; magnification: ×400).

To assess the role of inflammatory cells infiltration in sesame oil-associated anti-inflammatory effect, leukocyte count in BALF and histological examination were assessed. Sesame oil significantly decreased BALF cell count (1.72 ± 0.18) than in OVA-treated mice (2.56 ± 0.29) (Figure 5(a)). Sesame oil decreased inflammatory infiltration in the lungs from OVA-treated mice (4.57 ± 0.79) compared with that in OVA-alone group (8.43 ± 0.79) (Figure 5(b)). Further, histological examination showed severe inflammation (arrows) in OVA-SO group compared with that in Sham group (Figure 5(c)).

### 3.5. Effects of Sesame Oil on IgE Production in OVA-Treated Mice

**Figure 6:**
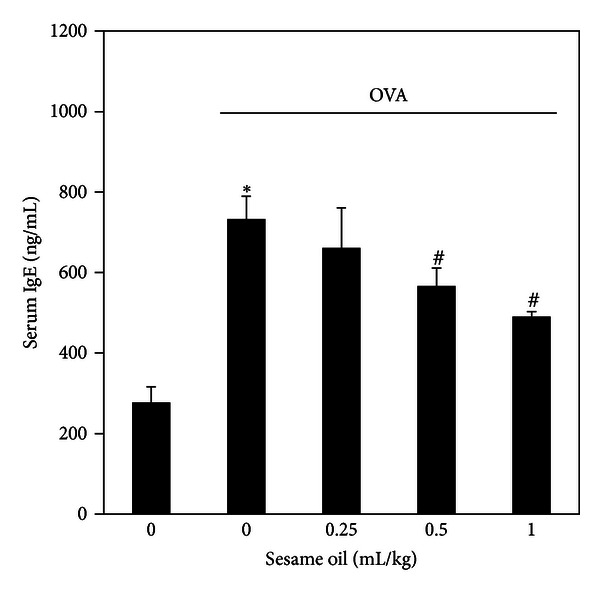
The effect of sesame oil on serum IgE levels in OVA-treated mice. Sesame oil (0, 0.25, 0.5, or 1 mL/kg, orally) was given to OVA-treated mice, and serum IgE level was determined. Data were mean ± SD (*n* = 8). Significant differences of measurement traits were analyzed using one-way ANOVA followed by Students' *t*-test. *P < 0.05 compared with control group; ^#^ *P* < 0.05 compared with OVA-alone group.

To examine the involvement of IgE in sesame oil-associated anti-inflammatory effect in allergic asthma, IgE level in serum was determined. The serum IgE level was significantly increased compared with control group. Sesame oil (0.5 and 1 mL/kg) (603.10 ± 77.58 and 550.67 ± 91.40) significantly decreased the IgE production induced by OVA (708.83 ± 61.84) (Figure 6).

## 4. Discussion

We, for the first time, demonstrated that sesame oil attenuated pulmonary disorder in an experimental allergic asthma. Sesame oil decreased pulmonary edema and inflammation, as well as neutrophil infiltration. Further, sesame oil decreased the IgE levels in OVA-treated mice. We suggest that sesame oil has the potential in treating patients with allergic asthma.

Sesame oil may decrease pulmonary edema by inhibiting pulmonary inflammation in our animal model. Although pulmonary edema is not a characteristic of asthma, it may occur during acute asthma exacerbation [23]. Proinflammatory cytokines, such as IL-1 and IL-6, amplify inflammatory response and increase the vascular permeability, both of which participate in the development of pulmonary edema in allergic asthma [4, 5]. In addition, nitric oxide produced by iNOS in pulmonary alveolar cell is also involved in allergic pulmonary edema [24]. Nitric oxide upregulates proinflammatory cytokine production and recruits leukocyte into inflammatory area [25–28]. In the present study, sesame oil significantly decreased pulmonary iNOS expression, nitric oxide production, and proinflammatory cytokine generation. It is likely that sesame oil attenuates pulmonary edema through inhibition of iNOS expression and subsequent nitric oxide and proinflammatory cytokine production, at least partially. 

Inhibiting leukocyte infiltration may be involved in sesame oil-exerted attenuation of pulmonary edema and inflammation. Leukocyte (including neutrophil, macrophage, and eosinophil) infiltration is commonly observed in case of allergic asthma [29]. Infiltrated leukocyte is a major source of proinflammatory mediator, such as cytokines and nitric oxide [30]. Inhibiting leukocyte infiltration shows a protective effect against pulmonary edema in various models [31, 32]. Sesame oil decreased cell counts in BALF and neutrophil infiltration in lungs. We suggest that sesame oil inhibits allergic asthma-associated pulmonary inflammation by inhibiting leukocyte, especially neutrophil, infiltration. 

Sesame oil may protect against allergic asthma by decreasing systemic IgE levels. IgE plays a central role in the pathogenesis and development of allergic asthma. The level of systemic IgE is positively correlated with the severity of clinical allergic asthma, although IgE has not been used as a clinical biomarker [33]. Inactivating IgE by antibodies shows a potent therapeutic effect against allergic asthma in both animal and clinical studies [34]. In the present study, the systemic IgE level was significantly lower in sesame oil-treated group. We suggest that inhibiting the release of IgE is associated with the protective effect of sesame oil against pulmonary inflammation and edema.

## 5. Conclusions

We summarize that sesame oil attenuates pulmonary edema and bronchial neutrophilic inflammation by inhibiting systemic IgE level in allergic asthma.
